# Reports of Cases Occurring in the Medical Ward of the Buffalo Hospital

**Published:** 1858-02

**Authors:** A. W. Nichols

**Affiliations:** Assistant to Chair of Clinical Medicine, University of Buffalo


ART. IV.—*Abstract of the Clinical Lectures delivered at the Buffalo Hospital of the Sisters of Charity, upon the Diseases treated in the Male Medical Ward, by* AUSTIN FLINT, M. D., *Attending Physician, during the present College Session, viz., from October* 14*th*, 1857, *to February* 27, 1858. Reported by A. W. NICHOLS, M. D., Assistant to Chair of Clinical Medicine, University of Buffalo.

No. III. *For December.*

*Bright's Disease.*

Two cases in which albumen was found to exist in abundance in the urine, for a considerable length of time, accompanied with more or less effusion into the areolar tissue, were presented to the notice of the class, during the month of December, and formed the subjects of the succeeding remarks by Prof. F. in several clinical lectures at the hospital.

The fact that cases of general dropsy, were not infrequently accompanied with an albuminous condition of the urine, was known some time prior to the discovery of Dr. Bright in 1827, and that a peculiar morbid condition of the kidney was often associated with the foregoing symptoms.

This discovery, though slowly accepted at first, has become so generally known to, and appreciated by, the profession, that not a few physicians at the present time, make no distinction between the mere presence of albumen in the urine and the disease called Morbus Brightii. The presence of albumen in the urine does not, however, necessarily denote the existence of this disease. It is only a symptom of disease, like jaundice, denoting the presence of some morbid process in the system.

When the albumen exists in great abundance and for a length of time, accompanied with general dropsy, then we may presume there is some pathological condition of the kidney present, more especially if there be anæmia coëxisting.

The symptoms of Bright's Disease are various, and many of them obscure, did we not possess our present knowledge of the pathology of this disease.

It may be well, then, to offer a very brief account of the pathology of this affection. Various appearances have been described by different writers, as occurring in the kidneys of those who have died with this disease. Dr. Bright, the discoverer of this lesion, describes the degeneration as peculiar, but divisible into three forms. Dr. Christison appears to take much the same view, regarding the disease, as "granular degeneration of the kidneys." Rokitansky, from the different morbid appearances of the kidney, divides the affection into no less than eight varieties. And lastly, Dr. Johnson, observing the microscopic appearances of the urine, makes five forms of disease associated with albuminuria. Thus, the subject is seen to lose much of its former simplicity, and to become quite complicated by the various observations of modern pathologists. There are objections, even if his views be correct, for adopting the refinements of Rokitansky; and no less objectionable is it to assume differences in disease, from the various results of microscopical examinations of the urine.

It is certain that the substance of the kidney undergoes some change incompatible with the healthy performance of its function. Histologists have not as yet, fully determined upon the minute anatomy of this organ. And although nearly all authorities adopt the views of Mr. Bowman in regard to the structure and physiology of the kidney, still the subject must be considered *sub judice*. Dr. Isaacs, of the University of New York, has quite recently submitted two papers on the "Minute anatomy and physiology of the Malpighian tufts," before the New York Academy of Medicine, which have been published in the transactions of that society. The opinions therein deduced from the author's microscopic examinations of these bodies, differ materially from the views of Mr. Bowman. Whilst it remains for future investigations to settle the various points under discussion, the present physiological views may be retained.

The opinions of Dr. Johnson, a recent writer on Diseases of the Kidney, are based, as before said, on the different microscopical appearances of the urine. He divides the disease into five forms; three of these, somewhat rare in occurrence, he terms *non-desquamative disease of the kidney*, in which no casts of uriniferous tubes are seen on examining the urine under the microscope; *fatty degeneration* of the kidney, in which there exists a great number of oil-globules in the uriniferous tubes; and *waxy degeneration* of the kidney, in which wax-like casts of the tubules are visible. The two remaining varieties are much more frequently met with, and are more interesting in reference to the microscopic appearances.

The first or *acute desquamative nephritis*, is regarded as an inflammatory condition of the kidney; in the urine, are seen "epithelial casts." The exudation which has been poured out into the uriniferous tubules has coagulated; and epithelial cells cast off from the sides of the tubules by a process analogous to that of desquamation, have become entangled in the coagulum, giving to the casts, their epithelial appearance; hence, the name, "epithelial casts."

The second variety is *chronic desquamative nephritis*, succeeding the acute; and characterized by what are termed "granular casts;" the granular matter being the epithelial cells disintegrated. As this form progresses, the epithelium disappears entirely, and the cast becomes of a "waxy" appearance. These waxy casts are of two kinds, large and small. Important inferences are derived from the size of these "waxy casts," as to the extent of the disease. If large, they denote an advanced stage of the disease, from the opinion that they have been found in tubules already deprived of their epithelial covering, by the process of desquamation, consequently showing that more or less of the cortical substance of the kidney has been rendered unfit for the secretion of urine.

It is sufficient, however, for our present purposes, to know that the peculiar morbid change or changes which take place in the secreting substance of the kidney, renders the organ more or less useless in the performance of its natural function. It produces, aside from the loss of the albumen of the blood, retention of the poisonous principles of the urine, of which urea has heretofore been supposed the most efficient.

But, anterior to the occurrence of any morbid change in the kidney, the disease no doubt involves some unknown alteration in the blood-constituents. This is shown, amongst other reasons, by the disease obeying the law of symmetry, as first remarked by Prof. F.: both kidneys being affected almost invariably, though one to a greater extent than the other.

To return to the symptoms, the connection of many of these with the morbid condition of the kidneys, is rendered quite plain, by the present pathological views. The symptoms of Bright's Disease may be considered as due to the effect of the loss of the albumen of the blood and the retention of the poisonous principles of the urine. In the category of symptoms referrible to the excessive drain of the albumen from the blood, may be mentioned, as follows: pallor of the face, due to anæmia; œdema, in the face, at first, usually; anasarca; pulmonary œdema; dropsy of serous membranes; smallness and feebleness of the pulse with not much, if any, acceleration.

The symptoms due to retention of the urea or other deleterious principles of the urine, are probably the following: epistaxis; amaurosis; vomiting and diarrhœa; neuralgia in different situations, especially in the forehead; cramps in the lower extremities; epileptiform convulsions; inflammation of the serous cavities, with effusion; coma, often sudden in its appearance.

The essential cause of the disease is as yet unknown. But, the affection in its acute form, very often follows scarlatina. In its chronic form, it is frequently found in persons who are addicted to the intemperate use of alcoholic liquors. It follows, also, exposure to changes of the atmosphere, more especially occurring during cold weather. It has been found to succeed intermittent fever, disease of the heart, and erysipelas. It occurs more frequently in males than in females; and is found at all periods of life, not excepting infancy.

The diagnosis of the disease, as far as regards the presence and amount of the albumen in the urine, is not difficult to determine in any case. The existence of a morbid change in the kidney, however, cannot so readily be predicted. For it has been seen that albumen may exist in the urine, without any lesion of the kidney; these being cases of albuminuria merely. But when the albumen continues to be present in the urine for a length of time, and in considerable quantity, especially if there coëxist general dropsy and persistent anæmia, there can scarcely be a doubt, that the patient is affected with Bright's disease.

Not a few cases of this affection, even where the morbid product has invaded to a great extent, the substance of the kidney, are overlooked by practitioners who pay very little, if any, attention to examinations of the urine. Such cases present symptoms which it is impossible to connect with the renal disease, by any process of reasoning *á priori.* And yet when, by examinations of the urine chemically and also by the microscope, it has been definitely determined that the patient is affected with Bright's disease; and still farther when we remember that in this disease, two grand events are constantly going on, namely, the outpouring of more or less of the albumen of the blood, and the retention of the urea or poisonous principles of the urine; then it is that we are able to connect the singular and otherwise unaccountable symptoms so commonly presented in Bright's disease with the morbid condition of the blood and of the kidneys in this affection.

The microscope finally is no inconsiderable aid in the diagnosis of this disease. Dr. Johnson, as before remarked, has based his divisions of albuminuria on the microscopic appearances presented in the urine. And whether more or less importance should be attached to his views, the study of these microscopic casts, in connection with the symptoms presented during life, and the condition of the renal organs after death, will doubtless lead to farther information in regard to the nature of Bright's disease.

As regards the prognosis and treatment, little need be said at present. The prognosis in the acute acute form of the disease, is generally favorable; although, there is great liability of the affection becoming chronic. When the disease is chronic, the prognosis is almost always unfavorable; although the unpleasant spmptoms of the disease may disappear for a time, eventually the atient will be carried off by some secondary disease.

The treatment in the acute form will be moderately antiphlogistic; being adapted to the particular case presented to our notice, always bearing in mind the tendency of this variety to become chronic.

When the patient is already laboring under the more advanced or chronic form of the affection, our remedial measures must still be suited to the individual case we are about to treat. If there be serous effusion in the form of anasarca or ascites, diuretics are most generally resorted to, and hydragogue cathartics and diaphoretics have sometimes been employed. Bitartrate of potassa is the form of diuretic very commonly used; and elaterium is the best hydragogue; as a diaphoretic, Dover's powder is probably the best.

After the above indication has been fulfilled, we may endeavor to diminish the outpouring of the albumen and consequently impoverishment of the blood. Gallic acid will be found highly useful as an internal astringent.

And with other remedial measures indicated in the particular case under treatment, tonics and sustaining diet, with attention to the preservation of an equable temperature of the surface of the body, together with exercise rightly managed, will constitute no inconsiderable part of a judicious and beneficial treatment of Bright's disease.

